# IMOS: improved Meta-aligner and Minimap2 On Spark

**DOI:** 10.1186/s12859-018-2592-5

**Published:** 2019-01-24

**Authors:** Mostafa Hadadian Nejad Yousefi, Maziar Goudarzi, Seyed Abolfazl Motahari

**Affiliations:** Department of Computer Engineering, Sharif University of Technology, Azadi, Tehran, Iran

**Keywords:** Aligner, Long read, Big data, Distributed processing, PacBio

## Abstract

**Background:**

Long reads provide valuable information regarding the sequence composition of genomes. Long reads are usually very noisy which renders their alignments on the reference genome a daunting task. It may take days to process datasets enough to sequence a human genome on a single node. Hence, it is of primary importance to have an aligner which can operate on distributed clusters of computers with high performance in accuracy and speed.

**Results:**

In this paper, we presented IMOS, an aligner for mapping noisy long reads to the reference genome. It can be used on a single node as well as on distributed nodes. In its single-node mode, IMOS is an Improved version of Meta-aligner (IM) enhancing both its accuracy and speed. IM is up to 6x faster than the original Meta-aligner. It is also implemented to run IM and Minimap2 on Apache Spark for deploying on a cluster of nodes. Moreover, multi-node IMOS is faster than SparkBWA while executing both IM (1.5x) and Minimap2 (25x).

**Conclusion:**

In this paper, we purposed an architecture for mapping long reads to a reference. Due to its implementation, IMOS speed can increase almost linearly with respect to the number of nodes in a cluster. Also, it is a multi-platform application able to operate on Linux, Windows, and macOS.

## Background

Long reads are usually very noisy having multiple substitutions, insertions, and deletions, c.f. PacBio RS II [1] and Nanopore MinION [2]. At the same time, long reads are valuable in bridging repeat regions which provide information not acquired by short reads. They can also be used to identify many structural variations such as translocations, duplication, etc. To exploit the full information embedded in such reads, one requires to align them accurately to the reference genome.

Several long read aligners are developed in the past. Each aligner has its own capability. The Pacific Bioscience, the company which makes PacBio RS II sequencer, presented BLASR [3] for aligning Pacbio long reads. It has good accuracy by reporting several locations with different scores for each read. Despite the costs (e.g. time, energy, storage) paid for finding every possible location, most of them have the least importance for downstream analysis due to the low alignment score. Heng Li presented Minimap2 [4] as the successor of his prior work BWA-MEM [5]. It is a very fast and accurate long read aligner. In addition to the great ideas including using minimizers [6], it is implemented very efficiently. Sedlazeck et al. [7] proposed a long read aligner which produces alignments optimized for structural variation detection. Meta-aligner [8] is designed based on statistical features of the reference genome. The main concern of Meta-aligner is to align all reads by respecting the integrity of them with no clipping. This conservative attempt makes it more accurate but rather slow in mapping. Therefore, we set an aim of this paper to improve both speed and accuracy of Meta-aligner in order to have a fast and accurate long read aligner not restricted to a specific OS.

With the ever-increasing size of genomics data and the growing demands for sequencing, ordinary single node software would not be able to satisfy all the computing needs where they last in order of days for a full run. Distributed processing as a cost-efficient solution to speed up the computations could be a good feasible choice in practice compared to other solutions such as using hardware acceleration (e.g. FPGA and ASIC). There are some aligners designed to work on distributed platforms. DistMap [9] is a distributed software deploying nine short read aligners on an Apache Hadoop [10] cluster. Abuin et al. developed two applications, called BigBWA [11] and SparkBWA [12] that distribute BWA on both Apache Hadoop and Apache Spark [13]. As mentioned by Abuin *et al* in [12], SparkBWA is the successor of BigBWA and provide a better performance. None of the prior works pay attention to hardware-aware optimizations. Another aim of this paper is to develop a working application for aligning long reads that outperforms current rivals and to present an efficient framework to build an application that could be easily upgradeable.

Meta-aligner developed in our lab uses genomes statistics to achieve higher performance. However, in its original form, it is not competitive to other aligners. Therefore, we propose some improvements that make it faster and more reliable in this paper. We also re-develop it and apply the improvements using Java. Although it can handle large datasets, it is also suitable for aligning small datasets without changing the OS. For the distributed working mode, we use Apache Spark, which is a sophisticated big data processing platform recently developed and widely adopted for large-scale applications. We propose and develop a distributed architecture for aligning long reads to a reference genome. We select our improved Meta-aligner(IM) and Minimap2 for deploying on the proposed framework.This shows that our framework is general and can be adopted by other aligners as well.

## Single node implementation

In our single node implementation, Meta-aligner is improved in five significant ways. In this section, we describe the details of improvements made on Meta-aligner. The first two makes the mapping more accurate, while the others three accelerates the program. Moreover, these upgrades also lead to an output more comfortable for downstream analysis. Then, we applied these improvements and implemented a new aligner in Java language. To the best of our knowledge, IMOS is the first long read aligner in Java. It is suitable for other contributors to use it in multi-platforms applications.

### Get feedback from the local alignment algorithm

**Fig. 1:**
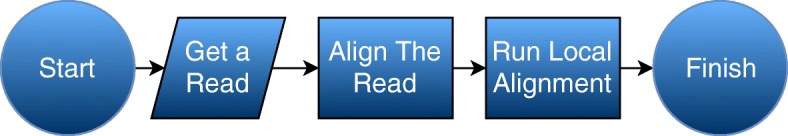
Meta-aligner: flowchart of computing procedure on a read

**Fig. 2:**
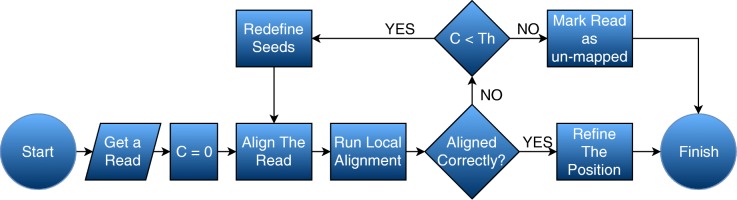
IMOS: flowchart of computing procedure on a read

We use feedback from the local alignment algorithm after mapping each read to gain higher accuracy by: (i) verifying that the edit distance is not more than expected from the input specification, and (ii) making the reported position more precise. Figures 1 and 2 show the flowcharts of computing procedure on a read for Meta-aligner and IMOS, respectively. Meta-aligner aligns all reads and then run local alignment. We changed the procedure by getting feedback from the local alignment algorithm which is based on the Smith-Waterman (SW) algorithm [14]. Consequently, we can compare the edit distance reported by SW and what we expect from input data to find out whether the read correctly assigned or not. Also, to avoid wasting time on the low quality or hard-to-map reads, we defined a threshold (Th) for the number of attempts at aligning a read.

Since in a long read, there are lots of insertions and deletions the reported position based on the information of seeds could be different from the exact position of the long read. For instance, a long read split into 20 seeds of length 40 base pairs. If we report the position of the long read based on the information of seeds 19 and 20, indels in 18 previous seed are implicitly ignored. Thus, the reported position might differ about 100 base pairs (15% of 18×40) from the exact position. Therefore, we use the information of the local alignment algorithm to refine the position.

### SureMap instead of bowtie

Meta-aligner used bowtie [15*] which considers no gap while mapping seeds to the reference genome. On the other hand, we used SureMap [*16] instead of Bowtie because it can handle indels for short reads. Therefore, SureMap can extract more accurate information from a seed. Hence, this improvement leads to better accuracy.

### Integrated design

The Meta-aligner structure has a top-level procedure that incorporates a prebuilt short read aligner in order to map seeds of a long read. These two parties communicate through storing input and output on the disk. The top-level procedure splits the reads into a number of seeds and writes them into several files. For each file, it calls the short read aligner with these files as input. Then, the short read aligner loads the input file from the disk to the main memory and writes down the output to the disk again. Finally, the top-level procedure loads the output file of short read aligner from the disk to the main memory and maps long reads with respect to the mapped short reads. This structure has two issues that degrade the performance of the mapping, I) high storage usage as a low-speed unit, and II) additive initialization time of the short read aligner with each call.

We re-implemented both SureMap and the top-level procedure in Java. By integrating them, they work as one unit in a same memory address space. This removes any storage or initialization overheads.

### Change traversing in the alignment stage

Meta-aligner has two main stages, alignment and assignment stages. In the alignment stage, it tries to find the position of a read by finding two seeds that mapped uniquely. If a read did not align in this stage, in the assignment stage, it tries to find the position of the long read regarding every possible position of every seed. Their results show that a large fraction of the dataset is mapped in the alignment stage with a shorter time per read and a small fraction have a long processing time per read in the assignment stage.

Meta-aligner traverses seeds with a brute-force algorithm. We use a randomized method to gain a higher speed. Our experiments show that two adjacent seeds have a high chance to be in a same region (random or repeat region). A random (repeat) region is a region or interval in the sequence of DNA that a read can (cannot) be uniquely mapped in it. Meta-aligner traverses seeds sequentially from first to last seed. Therefore, if a significant portion of a read located in a repeat region, Meta-aligner alignment stage will last long while computing seeds that are in repeat regions. We use a randomized method to gain a higher speed. Our traverse method has two states:

1. If the seed is in a repeat region, next seed will be one random seed among remaining seeds.
2. If the seed is in a random region, next seed will be one random un-traversed seed among adjacent ones unless both are traversed. In the latter case, next seed will be one random seed among remaining seeds.

Algorithms 1 and 2 show the traverse method of the original Meta-aligner and IMOS, respectively. It is evident that this improvement has no effect on accuracy and only increase the speed of the traverse method on average.


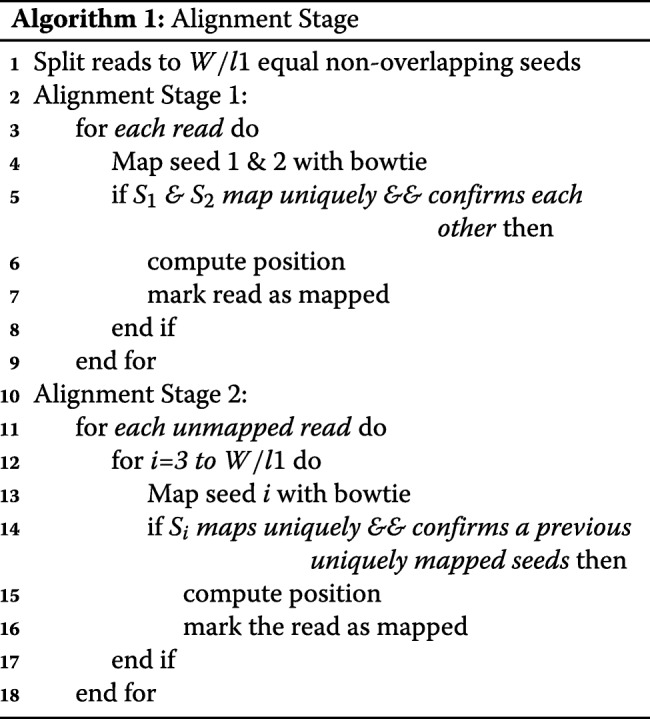



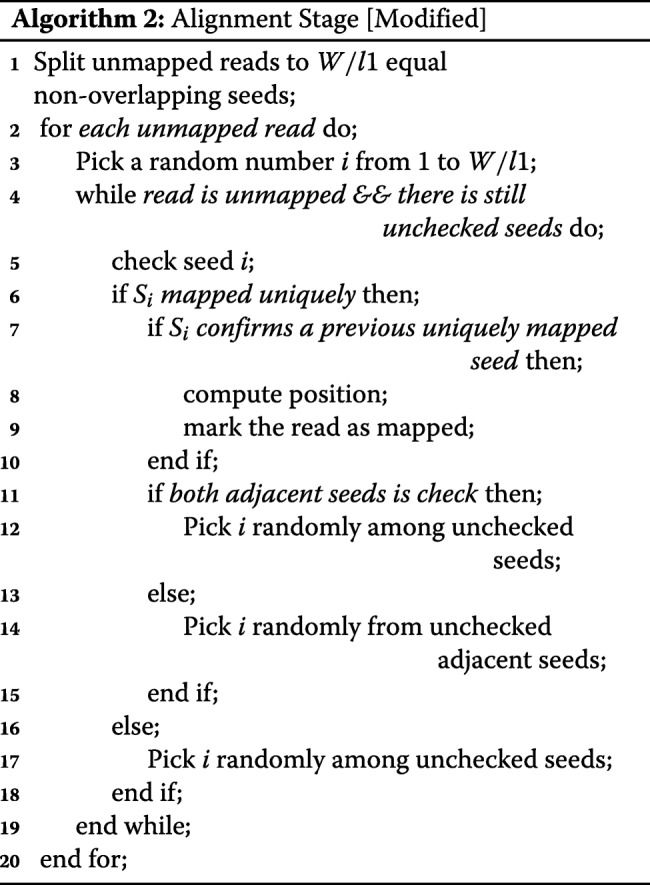


### Efficient search in the assignment stage

**Fig. 3:**
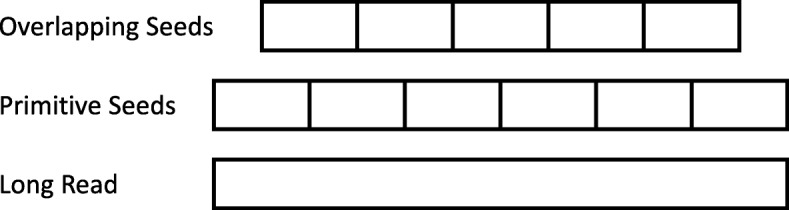
Meta-aligner fragmentation

After a read did not map in the alignment stage, the original Meta-aligner performs another fragmentation operation which splits a read into a number of seeds. In other words, it adds new seeds of the long read as shown in Fig. 3. The primitive seeds are the result of the first fragmentation done in the alignment stage and the overlapping seeds are the outcome of the new fragmentation in the assignment stage. The original Meta-aligner computes every seed (primitive and overlapping seeds) in its assignment stage. We do not process seeds that are not informative so as to gain a higher speed. Since primitive seeds computed once, they are less informative in the assignment stage. Also, It is possible to find two seeds that can be uniquely mapped among newly added seeds. Therefore, we only compute newly added seeds which are more informative.

## Distributed (Spark) implementation

In the best case, the performance of single machines can be doubled in every 1.5 years with respect to Moore’s law. Moreover, a two times faster machine is four times more expensive while distributed systems cost to performance ratio is almost constant. For having an unlimited (theoretically) processing power, we need a distributed system that allows us to double the performance every time by doubling the number of machines. Since our application works with a huge amount of data and processing of this amount of data lasts long, we need a distributed system to do jobs in a reasonable time.

In this section, first, the reasons why we chose Apache Spark and its introduction are presented. Our cluster design comes after that. Then, the technical implementation issues are explained and the solutions are presented. Afterward, an overview of our worker node design is presented. Finally, after discussing our batch implementation thoroughly, a summary of streaming implementation is presented.

### Platform

We had two directions I) implement our own platform, II) use a matured distributed processing platform. We chose the second option because:

- Implementing our own platform is time-consuming.
- It is difficult to implement a platform that considers every kind of failure.
- It is hard to relay on an immature distributer.

To determine which platform suits the application well, we consider the fact that aligning reads to a reference is intrinsically a batch job. Therefore, the platform should provide a high throughput in order to reduce the processing time. In addition, the runtime should scale almost linearly with respect to the number of nodes in the cluster.

After considering a number of different choices for deploying IM and Minimap2 in a distributed environment, we chose Apache Spark [13*] among Apache big data processing platforms [*10*,*^13^*,*^17^*–*19*]. It is an open source big data processing framework for distributed environments. It can work on a cluster of computing machines. According to results of evaluations done in [*20*,*^21^] which analyzed the performance of big data platforms through a number of standard benchmarks, Apache Spark provides better throughput and scalability in both batch and stream processing. As a summary, the main reasons for our choice are described as follows:

- **Open Source:** Spark is open source and has an active community which is growing and contributing rapidly.
- **Fast:** Spark is very fast and up to 100 times faster than its predecessors like Apache Hadoop [10] due to its implementation. It improves in many aspects such as storage and fault-tolerant mechanism.
- **Highly Scalable:** Spark tries to reduce runtime overheads in order to reach a linear speed up related to the number of nodes in the cluster.
- **Unified Platform:** It offers a wide range of services for different applications and implementations. In other words, it is an all-in-one platform. For instance, an application which is designed for batch processing can be re-designed for stream processing with a little effort. This greatly speeds up developing and maintenance of an application.
- **Easy to Develop:** There is no need to involve developers with low-level system controls such as managing the cluster. The developer needs only to concentrate on designing the algorithms and the program. This ease brings focus and concentration to developers for innovation.
- **Independent Design:** Each component of Apache Spark like the cluster manager can be customized independently.

**Fig. 4:**
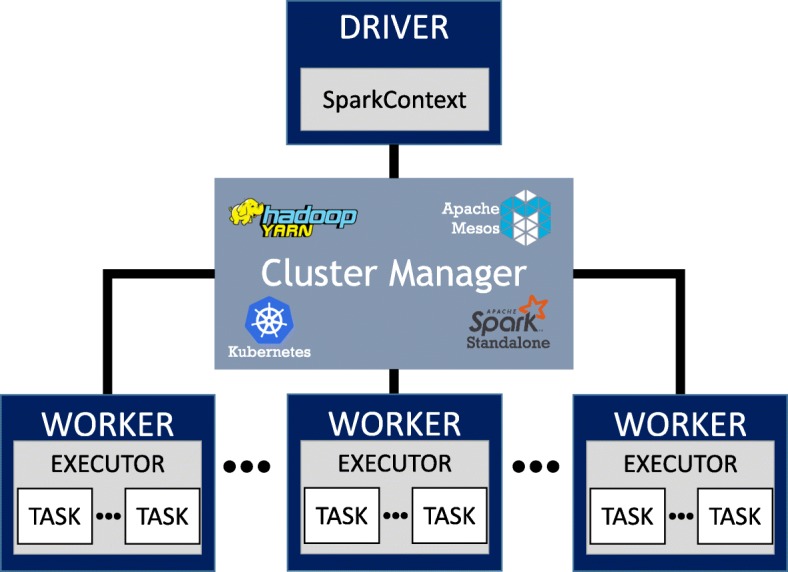
Apache Spark architecture

Figure 4 shows the architecture of Apache Spark which is master/slave. The driver runs the main function of applications and creates a SparkContext for each application which coordinates the independent set of processes of the parent application. The SparkContext can be connected to a cluster manager which could be one of Apache Spark Standalone, Apache Hadoop Yarn [22*], Apache Mesos [*23*], and Google Kubernetes [*24]. The cluster manager connects the master node to workers and allocates the required resources on the cluster to the application. Each worker node runs an executor process which is the unit responsible for computing and storing operations. It runs and coordinates tasks. Tasks are the smallest operational components that can be run in parallel. A set of tasks form a job and a set of jobs form an application. The SparkContext sends tasks to executors. Each rectangle is a component that could be placed on any networked machine. For instance, a driver and a worker can be placed on a same machine or two distinct machines.

Apache Spark can work with Hadoop Distributed File System (HDFS) [25] which is a highly fault-tolerant distributed file system. It is designed to work on low-cost commodity hardware. Similar to Spark, HDFS also has a master/slave architecture. An HDFS cluster has a NameNode as the master which manages the file system and controls the access to files. Each cluster may have a number of DataNodes as slaves that store a file as a number of blocks and execute file operations such as opening a file.

### Cluster design

**Fig. 5:**
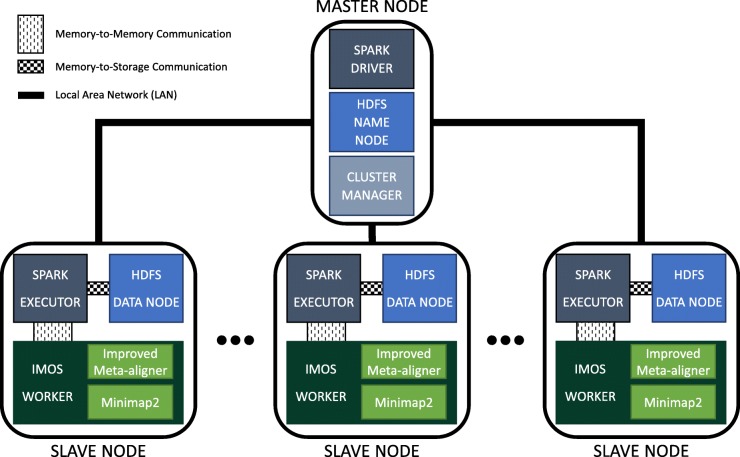
IMOS architecture. A master node (rounded rectangle) with a number of slaves ones (rounded square). Spark Driver, HDFS NameNode, and the cluster manager are placed on the master node. Each slave node contains a HDFS DataNode, a Spark Executor, and an instance of IMOS worker. Spark executor communicates with DataNode and IMOS worker through storage disk and socket as the medium, respectively. Components of Spark, HDFS, and our design are distinguished using slate gray, blue, and green colors, respectively

We implemented our improved Meta-aligner on the core Spark which is the batch processing engine of Apache Spark. Figure 5 shows the architecture of IMOS where the rounded rectangle and each rounded square represent a machine. Spark Driver, HDFS NameNode, and Cluster Manager which are the coordinators are placed on the master node. Since the coordinators do jobs such as scheduling and allocation periodically, it is better to have the master node near the workers. Therefore, we placed all of them on the same local area network (LAN). If a user wants to submit an application remotely, it is better to connect to the master node through SSH or something similar instead of placing the coordinators on the user machine. On every slave node, there are a Spark executor, an HDFS DataNode, and an IMOS worker which is an interface that can run either IM or Minimap2. The workflow of our design is such that the system first distributes the long reads among processing nodes using HDFS and then employs Spark to call the instances of IMOS worker where the data resides. Afterward, the IMOS worker runs an aligner among IM and Minimap2 to start mapping. Finally, The results can then be written on HDFS for being prepared for any downstream analysis or can be sent to the master node for being aggregated.

### IMOS worker

IMOS worker is designed to independent of the underlying aligner. It is an interface that bridges the Spark executor and the aligner. At the startup of the IMOS worker, the user defines its desired aligner. Then, the application runs the aligner in the modified mode as its child process. After the initialization has been done, IMOS worker starts listening to the Spark executor and takes the data with commands and passes them to the aligner. Finally, IMOS worker returns back the results as soon as the aligner’s job is completed.

In this paper, Meta-aligner and Minimap2 are modified to work with IMOS worker. Other aligners can be adopted similarly.

### Load balancing

Load balancing is the key to reach the full potential of a distributed system. The finish time of a distributed job is measured by the time that every task is done.

We presented a load balancer that works on top of a HDFS. Its core concept is to prepare the input file such that the characteristics of each chunk of data be similar when the HDFS distributes them across the cluster nodes. Therefore, there is no need to perform any change in the HDFS NameNode. Moreover, this can be done independently as a data preparation step. Thus, the running time of load balancing is not an issue since it is a static task. However, we provided a linear greedy solution that works near optimal.

Our analyses show that the two features of a FastQ file which have the most influence on the processing time are the average length and the number of reads. In addition, these two features can be extracted easily. As a big picture, we scan the FastQ file and group every *N* reads where *N* is the number of nodes in the cluster. After the creation of each group, the reads will be sorted by the length. Then, we distribute them one-by-one in a Round-Robin manner to the nodes such that a node receives the read with maximum length once in every *N* distributions.

Since the whole FastQ file consisting *R* reads is needed to be scanned and there is a sort with the order of *N* log*N* for every *R*/*N* groups, the time complexity of the load balancer is *O*(*R* log*N*). As the number of nodes is much smaller than the number of reads, it can be considered as a constant and be neglected.

### Implementation issues

We faced some serious concerns while trying to implement our design efficiently. These concerns are explained in the following.

#### Minimize storage usage

The key to Spark performance is its in-memory processing technique. It keeps track of data through a number of operations by storing the intermediate data reliably in the main memory. Accordingly, to conform the Spark architecture, the first question is how to implement our design to minimize the usage of storage as a low-speed unit in the system. One of the best high-performance designs for this application is that the reference files remain in the main memory for the entire application runtime to avoid storage-to-memory communication overheads. Apache Spark provides two type of variable for this purpose: *Broadcast Variables* and *Accumulators*. Both have problems that make them inappropriate for our design. *Broadcast variables* are broadcasted to every worker nodes in the application initialization. This has four main issues:

- **High Network Usage:** Broadcasting this large amount of data over the network will use the network inefficiently.
- **Initialization Time:** For each application, there will be an initialization time to broadcast the reference files.
- **Node Failure:** Each time a node faces a failure, the data must be broadcasted again.
- **Memory Overhead:** In Spark implementation, a broadcast variable is accompanied with a large meta-data. Our experiments show that an 11GB variable at the host occupies 17GB of the guest main memory.

On the other hand, *accumulators* can only be defined as primitives such as Integer. Hence, none of these options solve the problem of maintaining the reference files in the main memory. Therefore, we used another strategy.

Since the single node implementation of almost every aligner keeps the reference files in the main memory for the whole runtime, we decided to place an instance of the IMOS worker in every worker nodes as an independent application working with spark through a memory-to-memory medium. We chose socket programming on localhost as the safest medium which also provides an ultra-fast communication speed.

In this implementation, an instance of the IMOS worker runs at the worker node setup-time once at the cluster setup. The Spark executor, IMOS worker, and the selected aligner communicating through socket programming while the only storage usage is loading the data from HDFS by the Spark executor. In addition to solving the aforementioned problems of *broadcast variables* and *accumulators*, this design benefits from I) high-speed memory-to-memory transactions instead of using storage disk as the medium, II) the initialization of the aligner done once for multiple jobs.

#### Data structure

The second concern is about the data Structure. What data Structure is provided by Apache Spark and how to use it most efficiently. Apache Spark core concept is Resilient Distributed Datasets (RDD) which is a distributed collection of data that can be operated in parallel. RDD is not the data, it contains a number of partitions storing data. Further, an interesting feature is that it recovers automatically from failure. An RDD can be created by parallelization of a dataset in the driver program or loading from a file system like HDFS. In our design, an RDD is created by loading the input FastQ file from HDFS.

A possible question is that why partitioning is needed. Loading every record from the file system into a single partition requires a large amount of space in the main memory, but with this technique, records can be transferred to the main memory in a partition-by-partition quantization. Since the processing power is limited which obviously cannot process every record in parallel, it is enough to read a sufficient amount of data each time to make sure that the CPU is fully utilized. In our design, the partitions size is set to 64MB which is equals to HDFS block size. This size setting avoids repartitioning in order to save time. Moreover, 64MB is approximately the required size to store 4750 long reads with the average length of 7000 base pairs which is large enough to ensure that the CPU always has reads to process. Of course, this only affects the efficiency of memory usage with negligible effect on CPU time.

RDDs can perform two sets of operations: Transformations and Actions. Transformations create a new RDD from an existing one. Actions collect and return the values stored in the RDD to the driver program. All transformations are lazy which means they will not compute anything until an action requires the result of them. This infers that there is no need to worry about memory usage while chaining a number of transformations.

In our design, IMOS should create an RDD containing reads from HDFS. Then, it should send read mapping task with a transformation and return the mapped information with an action. There are two suitable transformations for read mapping: *map()* and *mapPartitions()* methods. The former operates on elements of the RDD while the latter operates on a partition (list) of elements from the RDD. We have decided to choose *mapPartitions()* because of two reasons:

- **Reduce Overheads:** As mentioned earlier, we need to send reads from Apache Spark executor to the aligner through a localhost connection. Sending reads in burst could use network efficiently. Still, An ideal high-performance design is to keep CPU always working at maximum utilization. Using batch data reduces I/O overheads leading to a better CPU utilization.
- **Custom Thread Control Mechanism:** Using *map()* method, Spark creates a thread for each read. Creating and killing threads in a high number would waste a lot of time. Furthermore, Spark may create more threads than the number of physical cores which is not recommended for a high-performance design due to the race for shared resources and the high number of context switches. It is noteworthy that there are also better opportunities for maximizing CPU usage by customizing threads when reads arrive in batch.

#### Controlling threads

As stated above, we need to control the threads. So, the questions are “Which implementation of threads suits our design?” and “How to implement threads to work efficiently together providing mutual exclusion as there are many shared resources?”. We designed our own thread control mechanism. Instead of creating a thread for each read, we create a number of threads equal to the number of cores and assign each thread to a core. Moreover, we stepped farther and used Java Thread Pool instead of the ordinary threads. It enables us to use wake/sleep instead of create/kill to save time. The *mapPartitions()* method gives a list containing the reads. We divide the list into the number of threads and assign each part to a thread. For maximizing the CPU usage, every thread must start and end together. Therefore, we need load balancing to maximize utilization. We distributed reads with respect to the length of reads such that the average read length of every part is almost equal. Since our distribution is not perfect, we designed a race-free double end queue for storing the reads that are assigned to each thread. When a thread finishes the processing of its own queue, it joins a thread which has more remaining reads in its queue.

Spark may call *mapPartitions()* method more than once at the same time. If we create a number of threads with each call, there might be more threads than the number of cores. As we discussed earlier, this is not pleasant for a high-performance design. So, used a semaphore-based mechanism to ensure that only one partition is being processed at each time.

### Worker node design

**Fig. 6:**
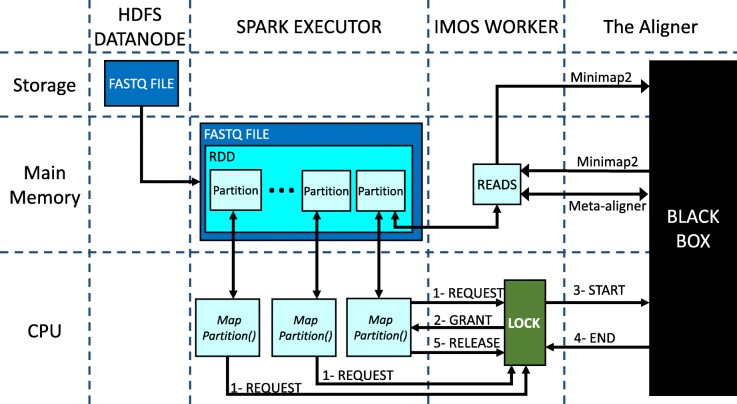
Worker node workflow. Horizontal and vertical line separates different hardware and software units, respectively
- **Spark Executor:** The spark executor works as a manager to divide the chunk into a number of partitions that formed an RDD and transfers them to the aligner for being processed. It also calls a *mapPartitions()* function on each partition.
- **IMOS Worker:** It is an interface between the Spark executor and the aligner. There is also a lock to control the parallelization of the *mapPartitions()* functions.
- **Aligner:** It is shown as a black box since it could be any aligner.

Figure 6 shows the workflow of each worker node in our design. As it is shown, there are four entities with separate memory address space:

- **HDFS DataNode:** The only entity that always uses storage is DataNode that stores a chunk of the input FastQ file.

The Scenarios for the cases that either the IM or Minimap2 is selected as the aligner are a little bit different where we will elaborate more in the sequel.

First, the Spark executor reads the chunk from HDFS. For the IM case, it is the only storage-to-memory communication. The Spark executor stores the FastQ file as an RDD in the main memory. The RDD contains a number of partitions where each of them is a list that contains a part of the reads. As mentioned above, a *mapPartitions()* function is called on each partition. Each *mapPartitions()* sends a request to the IMOS worker to get the lock and use the aligner. The lock grants functions in First-Come-First-Served order like a queue. Then, the granted function transfers the list of reads that stored in the partition to the memory space of IMOS Worker. Unlike the IM case that IMOS worker passes reads through socket-programming, IMOS worker uses storage in the Minimap2 case. After the transfer is completed the IMOS worker calls the aligner to start mapping the reads. When the mapping process is finished, the aligner returns the results to the IMOS worker. Similarly in both cases, returning the results goes through a memory-to-memory medium. Afterward, the granted function gets the result back from IMOS worker and releases the lock. Finally, the granted function can send the results to the Spark Driver or store them on the HDFS DataNode. This procedure repeats until every partition in every RDD is processed.

### Apache Spark streaming implementation

Last but not least, we also implemented our improved Meta-aligner on the Spark Streaming. The Spark streaming is an extension to the core Spark. It can receive streams from brokers such as Apache Kafka [26]. The streams arrive in a record by record quantization. In every specified time interval, the spark streaming gets all the arrived records and organize them together in a bundle called micro-batch. Each micro-batch will be passed to the core Spark and treated like a normal batch. The communication and processing time could overlap each other by using stream processing techniques. Therefore, at least theoretically, it could reduce the latency and total processing time of each dataset. We discussed it with more details in the Discussion section.

## Results

### Experimental setup

We performed two sets of experiments to evaluate the performance of IMOS in single node and distributed modes. In the single-node, we have used a server with a Intel Xeon E5-2630 v3 processor having 8 cores and 32 GB of RAM operating on Ubuntu 14.04 LTS. We used Apache Spark 2.2.0 for both batch and streaming experiments. In the distributed mode, we deployed an apache spark cluster of 10 nodes with one driver node and 9 worker nodes. Each node has a configuration similar to the one used in the single node.

### Datasets

Table 1 presents characteristics of eleven datasets used to examine the performance of aligners. Nine of them are synthetic designed for evaluating speed and accuracy for different traits using three different read simulators: Wgsim [27*], SimLoRD [*28*], and PBSim [*29*]. We used Wgsim to generate reads with different length and error rate. Its Synthetic datasets designed in two classes: short reads (Wgsim-S-class) and long reads (Wgsim-L-class). Although IMOS developed especially for high error long reads, we also evaluate the performance of aligning short reads too. SimLoRD and PBsim are used to simulate the PacBio SMRT sequencing. SimLoRD is used with its default parameters which are driven from public PacBio datasets and PBsim is used with its sampling-based method to produce reads similar to the real datasets used in this paper. We used two real datasets that are samples from Human 54 × and Han Chines Trio PacBio reads with accession numbers of SRX533609 and ERX1366175 published in NCBI Sequence Read Archive [*30]. They are used for measuring speed on a real dataset. Note that, the reference genome used in all experiments is human genome hg19.

**Table 1:** Datasets Characteristic: The name of synthetic datasets contains the read simulator name

DataSet name	Dataset size (MB)	Read length (bp)	Read length range (bp)	Mismatch (%)	Indel (%)	Number of reads
Wgsim-S0	59	300	FIX	0.9	0.1	100000
Wgsim-S1	193	1000	FIX	0.9	0.1	100000
Wgsim-S2	193	1000	FIX	0	10	100000
Wgsim-S3	193	1000	FIX	9	1	100000
Wgsim-L0	232	7000	FIX	1	16	20000
Wgsim-L1	232	7000	FIX	0.9	0.1	20000
Wgsim-L2	458	12000	FIX	1	16	20000
SimLoRD	315	8182	500-34687	1	16	20000
PBSim	343	7596	181-24998	1	16	22556
SRX533609	2589	6890	500-39445	1	16	174537
ERX1366175	800	12997	503-55908	1	16	30713

For Wgsim datasets, There are two type of synthetic datasets, S- and L-class which refers to Short and Long reads and their name start with S and L, respectively. The last two datasets are real and represent by the accession number

### Single node experiments

We analyzed the performance of our Improved Meta-aligner, the original Meta-aligner, BWA-MEM, and Minimap2. Comparison of Improved and the origianl Meta-aligner demonstrate how effective are our improvements and that of Minimap2 and BWA-MEM helps the better comparison of IMOS and Spark-BWA. Table 2 shows the performance of the tools for every dataset. We filter out alignments with MAPQ less than 10 and then measure the accuracy and map rate. Our measurement of accuracy is the number of SAM file [31] lines with the correctly reported position to the number of all lines. Map Rate is the percentage of mapped reads (containing incorrect ones) to all.

**Table 2:** Single Node performance comparison of IMOS and the original Meta-aligner

Dataset	Tool	Map rate (%)	Accuracy (%) *T*=0	Accuracy (%) *T*=10	Accuracy (%) *T*=100	Time (s)
Wgsim-S0	IM	99.66	94.5	94.9	94.9	40
	Meta-aligner	95.31	93.40	93.73	93.74	58
	BWA-MEM	97.81	99.81	99.99	100	20
	Minimap2	97.44	97.94	99.99	99.99	12
Wgsim-S1	IM	99.95	97.36	97.9	97.9	97
	Meta-aligner	97.79	94.76	96.27	96.29	174
	BWA-MEM	98.89	99.78	99.99	99.99	79
	Minimap2	98.67	97.86	99.99	99.99	25
Wgsim-S2 (Indel rich)	IM	99.43	87.17	95.52	96.62	360
	Meta-aligner	70.74	21.58	79.6	90.65	501
	BWA-MEM	97.65	85.42	99.94	99.97	130
	Minimap2	97.99	75.76	99.16	99.98	23
Wgsim-S3 (Mismatch rich)	IM	99.46	95.13	96.62	96.62	352
	Meta-aligner	99.05	67.09	96.21	96.23	202
	BWA-MEM	98.38	82.18	90.86	99.91	134
	Minimap2	97.79	76.60	98.94	99.99	24
Wgsim-L0 (Indel rich)	IM	99.96	78.04	97.27	97.37	780
	Meta-aligner	79.96	7.41	39.2	92.37	1928
	BWA-MEM	98.84	74.78	99.69	99.98	180
	Minimap2	99.04	59.02	94.58	99.98	47
Wgsim-L1	IM	99.81	99.02	99.05	99.05	438
	Meta-aligner	99.49	90.71	97.71	97.73	2306
	BWA-MEM	99.20	99.10	100	100	118
	Minimap2	99.05	98.06	100	100	33
Wgsim-L2 (Indel rich)	IM	98.63	76.61	99.14	99.14	1954
	Meta-aligner	95.97	5.02	31.56	94.23	6103
	BWA-MEM	99.22	75.00	99.77	99.98	318
	Minimap2	99.22	58.69	94.74	99.98	72
SimLoRD	IM	92.79	89.75	93.69	93.69	991
	Meta-aligner	92.79	0.08	22.15	42.09	1851
	BWA-MEM	92.43	85.46	95.98	97.65	458
	Minimap2	92.43	79.88	98.07	99.89	75
PBSim	IM	99.75	88.60	95.81	95.96	1098
	Meta-aligner	90.55	1.05	5.15	40.49	3918
	BWA-MEM	98.65	65.22	96.01	97.65	567
	Minimap2	98.93	54.50	95.56	99.98	78
SRX533609	IM	92.35	-	-	-	9031
	Meta-aligner	70.05	-	-	-	24,393
	BWA-MEM	92.75	-	-	-	3653
	Minimap2	89.58	-	-	-	348
ERX1366175	IM	97.33	-	-	-	2780
	Meta-aligner	88.15	-	-	-	14,303
	BWA-MEM	95.24	-	-	-	1512
	Minimap2	94.14	-	-	-	146

Whereas for the synthetic datasets we know the real position of each read, we calculate the accuracy by comparing the real position with the one that the tool declared. The parameter T which is illustrated in the Accuracy column is for Tolerance. Suppose T, P, and X as Tolerance, the real Position, and the declared position. if *X* satisfies in the equation *P*−*T*<=*X*<=*P*+*T*, then the declared position is correct.

Since the actual position for the real dataset is not known, calculating accuracy is not possible. We could have calculated the accuracy by edit distance factor. If an aligned read is more than a certain percent similar to the reference, then count it as correctly mapped read. A significant concern with this calculation is the fact that due to high error rates, a read may be aligned to a position different than the real one by chance. These types of errors can directly affect the correctness of any downstream analysis.

The results confirm the improvements. IM always performs better than the original Meta-aligner in terms of Map Rate, Accuracy, and Speed. There is an exception that IM is slower than Meta-aligner for Wgsim-S3 dataset where mismatches and indels happened frequently and rarely, respectively. This happened because the original Meta-aligner uses Bowtie which is more efficient in handling mismatches than SureMap. The point is that IM can offer better accuracy even in this case. However, it is noteworthy to mention that the Wgsim-S3 is an unusual case especially for PacBio datasets. Comparing results of Wgsim-S-class and Wgsim-L-class datasets, we can conclude that IM works better at least under one of these circumstances: 1) Read Length is very long, 2) Error rate of read is very high. Despite we designed IM for long noisy reads, the result shows a little difference between the result of S-class and L-class. This is interesting as it shows the robustness of IM. In terms of speed, IM is up to 6 × faster than Meta-aligner.

Moreover, the comparison of IM with BWA-MEM and Minimap2 shows that IM is competitive in term of map rate and accuracy. The forth column of Table 2 shows that IM always has the best accuracy for indel rich datasets where *T*=0. A main reason for the better speed of BWA-MEM and Minimap2 w.r.t IM is the programming language used for the implementation. Although Java is platform independent, it is slower than C ∖C++.

As we expected, Minimap2 is always faster than BWA-MEM. Specially for PacBio reads, it is about 10 times faster. Although it offers slightly better accuracy for Pacbio read, it has similar accuracy for other cases.

The real datasets characteristic are like PBSim and SimLoRD. We can approximate the accuracy of the tools by the results of these two. We can conclude that IM accuracy is always better than Meta-aligner alongside higher map rate for real datasets and is competitive with BWA-MEM and Minimap2 where it is better for smaller *T*s and a bit worse for bigger *T*s. Given our definition of accuracy, one can conclude that IMOS output is trustworthy for any downstream analysis.

### Apache Spark experiments

IMOS also is designed and implemented as an Apache Spark batch as well as streaming application to be executable on clusters. As mentioned before, our rival in the distributed environment is SparkBWA. Usually, a Spark cluster has a master node with a number of worker nodes. Since an instance of IMOS single node should be run on each slave node, the computational process remains the same. Therefore, accuracy and map rate for each dataset was the same as the single node experiments.

**Fig. 7:**
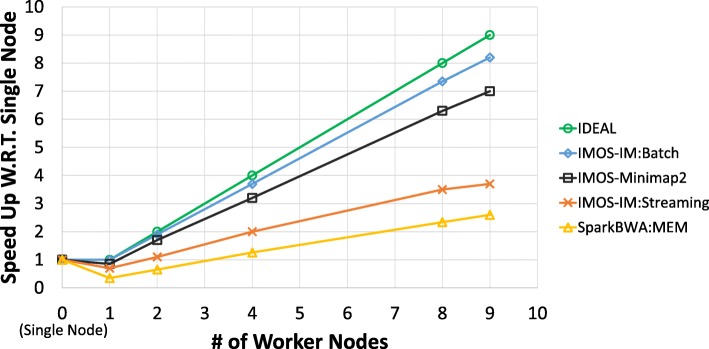
Scalability of application on a spark cluster. Zero as number of worker nodes is the results of single node with no spark involved

We deployed tools on an apache spark cluster of 10 nodes, one master and nine workers. The master node is placed at the middle of a star topology connected to nine other worker nodes. We repeated the experiment for different number of worker nodes to evaluate the scalability of tools. Figure 7 shows the speed up of tools with single node performance as the reference point. As expected, the performance of each tool almost doubles with doubling the number of nodes. Therefore, the relation ratio of tools approximately remains constant. Hence, selecting this reference point matters because the effect of implementation overheads is more recognizable. Comparison of worker node numbers of 0 (single node) and 1 shows the overheads of distributed implementation. The results show that IMOS with IM as aligner in batch mode has the least overhead and with Minimap2 has slightly more overheads because it uses storage. Unlike IMOS that keeps the aligners data in the memory for entire application runtime, SparkBWA calls BWA for each partition meaning initialization and more importantly loading reference files should be run for each partition. This is the major factor of high SparkBWA overheads alongside use of storage as interconnect media between Spark executor and BWA.

IMOS with IM and Minimap2 speed up is about 2.5 × and 3 × better than SparkBWA. Moreover, according to results of Table 2, Minimap2 is about 10 × faster than BWA-MEM. Therefore, IMOS is roughly 25 × faster than SparkBWA when using Minimap2. In addition, it is fascinating to know that although IM is about 2 times slower than BWA-MEM, IMOS even with IM is faster than SparkBWA (about 3/2 times). The results of streaming implementation are not as expected and is discussed in the Discussion section.

**Fig. 8:**
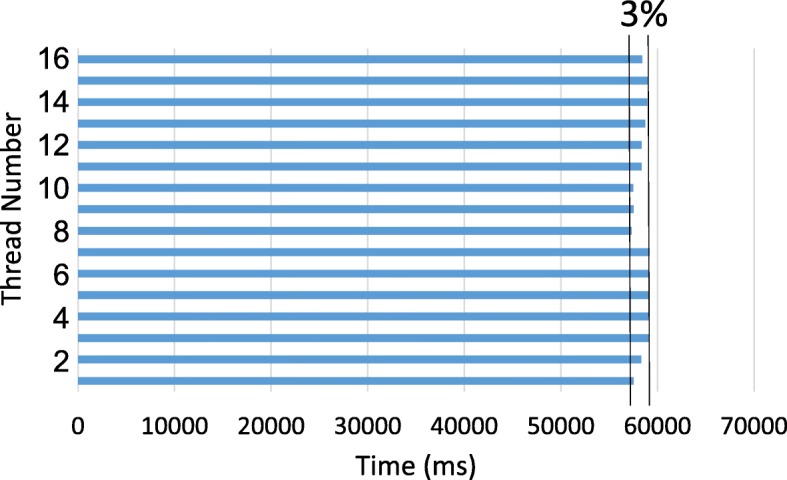
Processing time of 16 threads for a partition of 1000 reads

We also analyzed the performance of our thread control mechanism used in IMOS-IM. Figure 8 shows the processing time of each 16 threads in the thread pool for a partition containing 1000 reads. Note that the result for every other partition was very similar, so we only present an example. As shown, the maximum difference between the processing time of threads is only 3%. This shows the effectiveness of our load balancing and thread control mechanism. The average CPU utilization with this technique was about 99%.

## Discussion

In this section, we first compare choosing between batch and streaming processing. Then, we report the bottleneck analysis of IM and a future solution.

### Batch vs streaming

The input data of this application is a Fasta file containing reads. This file can be very huge in size. Therefore, the communication time is not negligible. For instance, a Fasta file containing 10 × coverage of human genome which counts as a low coverage would be 60GB. Transferring this file through almost the highest Internet connection which is reported to be about 184 Mb/s according to Akamai [32] will last about 45 min. Stream processing is able to overlap the communication time with processing time. So, we implemented IMOS-IM using Spark Streaming. As shown in Fig. 7 scalability of spark streaming is not as good as its batch processing. Our analysis shows that the micro-batch mechanism used for spark streaming and its scheduler for distributing data are the two main overheads. Also, if the system is fully utilized then the communication time of next job overlaps with the execution of the current job. Therefore, seeing the whole system as a pipeline, we can only count the execution time for each dataset which is the dominant time. Thus, we decided to release IMOS working with Apache Spark batch processing.

### Bottleneck analysis

We have analyzed the performance of IMOS-IM on a single node in order to find the bottlenecks. We have profiled CPU, cache, main memory, storage, and context switches when the mapping was running. Our analyses are presented for each item in the following.

- **CPU:** The average reported CPU utilization over the application runtime is 98% approximately which verify the maximum CPU utilization.
- **Context Switches:** As expected, since we do not create more threads than the number of cores, the context switch occurs rarely.
- **Storage:** We monitored the hit rate of main memory. The hit rate only falls with the arrival of new partitions. This means that the storage usage is just as expected.
- **Main Memory:** We monitored the read and write speed of the main memory. Summation of read and write speed never reaches the maximum throughput of the used RAM. This reveals that the main memory is not the bottleneck. However, the high amount of data read and data write between CPU and RAM gives us a clue that the cache may not function properly.
- **Cache:** We analyzed the hit rate of cache L3. Its hit rate is 40% approximately. As we expect the hit rate of L3 cache to be more than 50%, the cache has the highest chance to be the bottleneck.

To verify that cache is the bottleneck, First, we split our work into two major part, I) Finding the position of a read using Suremap [16] and II) Local alignment using the Smith-Waterman algorithm [14]. Timing analysis shows that finding position scales almost linear with the number of cores, but local alignment does not. Our analysis shows that the bottleneck for local alignment is low cache L3 hit rate which is about 15% on average that prevent local alignment as well as the whole program to scale linearly on a single node. Note that 15% for the hit rate of L3 cache is extremely small. Therefore, using a more scalable local alignment algorithm could lead to a system that scales linearly as Altera [33] implemented the Smith-Waterman algorithm using FPGA and gained around 200x speed up. Consequently, using FPGA as an accelerator could make the local alignment time negligible and and improve the overall performance.

## Conclusion

We have presented IMOS (Improved Meta-aligner and Minimap2 On Spark) which is a long read aligner suitable for noisy Pacbio reads able to be run on a single node (IM) as well as on an Apache Spark cluster (IMOS-IM and IMOS-Minimap2 respectively representing our port of IM and Minimap2 to our IMOS framework). One of our main intentions was to improve Meta-aligner in both accuracy and speed. We have improved the original Meta-aligner in 5 significant ways and re-implement it in Java. To the best of our knowledge, IMOS is the first long read aligner implemented in Java able to run on Linux, Windows, and macOS easily. IMOS is up to 6 times faster than the original Meta-aligner. Still, IMOS has a higher map rate alongside a higher accuracy. Eventually, the result of IMOS is trustworthy for any downstream analysis.

The other intention of ours was to design a framework suitable for mapping genomic sequences to a reference genome in a distributed environment. We ported both IM as well as Minimap2 to our IMOS framework. Moreover, we provided an interface to be able to run other aligners as well. IMOS is up to 25 × and 1.5 × faster than the current best-performing distributed aligner on Spark, SparkBWA, when with Minimap2 and IM as aligner respectively.

The direction for our future works is to integrate our design with a downstream analysis like structural variation detection to use the benefits of integration. Designing both upstream and downstream analysis together provides more optimization opportunities because one can design each side to work better with the other side.

## Availability and requirements

**Project name:** IMOS

**Project home page:** https://easy.ce.sharif.edu/imos

**Operating system(s):** Platform independent

**Programming language:** Java and C

**Other requirements:** Java 8, Apache Spark 2.1 and later, Apache Hadoop 2.7 and later

**License:** CC BY 4.0.

**Any restrictions to use by non-academics:** none

## Abbreviations

CPU: Central processing unit
GB: Gigabyte
HDFS: Hadoop distributed file system
IM: Improved meta-aligner
I/O: Input/output
LAN: Local area network
MB: Megabyte
Mb/s: Mega bit per second
RAM: Random access memory
RDD: Resilient distributed dataset
SW: Smith-Waterman
Th: Threshold
